# A Comparative Outcomes Study: Is Routine Hip Abduction Bracing Necessary After Gluteus Medius Repair?

**DOI:** 10.7759/cureus.116302

**Published:** 2026-09-15

**Authors:** Saavan S Boghara, Veshal Masih, Andrea H Johnson, Jane C Brennan, Benjamin M Petre, Justin Turcotte

**Affiliations:** 1 Orthopedic Research, Anne Arundel Medical Center, Annapolis, USA; 2 Physical Therapy, Anne Arundel Medical Center, Annapolis, USA; 3 Orthopedics, Anne Arundel Medical Center, Annapolis, USA; 4 Orthopedic Surgery, Anne Arundel Medical Center, Annapolis, USA; 5 Orthopedic and Surgical Research, Anne Arundel Medical Center, Annapolis, USA

**Keywords:** arthroscopic gluteus medius repair, clinical outcomes, gluteus medius tear, hip brace, physical therapy

## Abstract

Introduction

Postoperative protocols regarding hip bracing after the surgical repair of a gluteus medius tear remain variable. It is unclear whether hip bracing improves short-term outcomes in patients undergoing gluteus medius repair. The purpose of this study was to evaluate the combined six-month complication and reoperation rate before and after discontinuing postoperative hip brace use after a gluteus medius repair.

Methods

A retrospective review of 162 patients undergoing gluteus medius repair by a single, fellowship-trained surgeon was performed. The no-brace protocol was implemented in October 2020. Prior to October 2020, all patients were prescribed a postoperative brace, and this served as the historical control group. Starting in October 2020, the postoperative brace was no longer prescribed, and this served as the study group. The remainder of the perioperative protocol was the same for both groups. All patients immediately began physical therapy and had toe-touch weightbearing restrictions for three weeks postoperatively. The primary endpoint was the combined complication and reoperation rate within six months postoperatively.

Results

The groups were similar demographically, with the only significant difference being that the no-brace group had a higher percentage of patients undergo open repair (29 (33.7%) vs. 14 (18.4%); p=0.028). There were no significant differences in outcomes at 30 days when looking at emergency department returns (2 (2.3%) vs. 4 (5.3%); p=0.421) and readmissions (0% vs. 2 (2.6%); p=0.219). At six weeks, there were no significant differences in falls (2 (2.3%) vs. 1 (1.3%); p=1.000) and other complications (1 (1.2%) vs. 2 (2.6%); p=0.601). Similarly, at six months, there were no significant differences in falls (4 (4.7%) vs. 1 (1.3%); p=0.372) and other complications including deep vein thrombosis, re-tear, fibromyalgia flare, and fracture (5 (5.8%) vs. 5 (6.6%); p=0.840). There was also no significant difference between groups in reoperation rates within six months (0% vs. 2 (2.6%); p=0.219). The combined six-month complication and reoperation rate was also not significant between groups (10 (11.6%) vs. 9 (11.8%); p=0.966).

Conclusion

In patients undergoing gluteus medius repair, postoperative bracing was not associated with differences in complication rates over the six-month postoperative period. Based on this result, discontinuation of postoperative bracing appears safe in this population. Further study is needed to replicate these results with a larger sample size.

## Introduction

Gluteus medius tears are a common cause of greater trochanteric pain syndrome, presenting with lateral hip pain and possible gait abnormalities [1]. The gluteus medius has been described as the "rotator cuff of the hip" with tears most commonly resulting from chronic degeneration, but acute traumatic tears are possible [2,3]. Excessive hip adduction and flexion increase the compression of the tendons against the greater trochanter, which may contribute to tears in the deep and anterior portion of the tendon [4]. Clinically, patients present with lateral hip pain aggravated by ambulation and tenderness over the posterolateral aspect of the greater trochanter. On physical examination, there will be weakness with hip abduction and internal rotation [5]. Women tend to suffer more from this issue than men which may be due to the anatomy and biomechanics of female hips, and it usually presents between 40 and 60 years [4-6]. Nonoperative treatments include physical therapy, anti-inflammatories, and corticosteroid injections [7]. Surgical intervention is indicated when there is loss of abductor power, there is Trendelenburg gait, and nonoperative management fails [1].

Surgical treatments of gluteus medius tears include open or arthroscopic repair. Recent literature has shown favorable outcomes with arthroscopic repair, leading to the increased adoption of this approach among surgeons [8]. Rehabilitation protocols following the surgical repair of gluteus medius tears include a progression in weightbearing and range of motion, with an initial avoidance of hip flexion beyond 90 degrees, adduction past midline, active internal rotation, and passive external rotation. These recommendations are general guidelines, requiring individualization depending on the tear severity and repair type [1,8]. Postoperative protocols on hip bracing after arthroscopy, however, remain controversial. Proponents argue that the brace helps offload the muscles to decrease pain and reduce the use of hip flexors while walking [8]. On the other hand, braces cost up to $800, and patients are often dissatisfied with them [9]. It has been reported that between 28% and 59% of surgeons prescribe postoperative brace use after surgery for femoroacetabular impingement (FAI). Among the surgeons who do recommend brace use, bracing protocols are variable in terms of the range of motion restrictions, and the length of bracing ranges anywhere from one to six weeks [10,11].

The aim of this study was to retrospectively evaluate patients undergoing gluteus medius repair before and after implementing a no-bracing protocol by examining outcomes at 30 days, six weeks, and six months following surgery; the primary outcome of interest is the combined complication and reoperation rate at six months postoperatively. We hypothesized that patients would have similar outcomes before and after the implementation of the no-brace protocol.

## Materials and methods

Perioperative protocol

All surgeries were performed on an outpatient basis under general anesthesia; regional anesthesia for postoperative pain control was used at the discretion of the surgeon and anesthesiologist. Prior to October 2020, all patients received a hip abduction brace to be worn for three weeks postoperatively. After this time, routine bracing was discontinued. All other postoperative protocols remained consistent over the study period, including physical therapy protocols, range of motion restrictions, weightbearing restrictions, and follow-up schedule. The postoperative rehabilitation protocol includes range of motion restrictions including no hip active abduction, no active or passive adduction, and extension for the first three weeks with gradual progression after that. Patients were toe-touch weightbearing for the first three weeks with gradual progression from four to six weeks to full weightbearing.

Data collection and analysis

All patients who underwent gluteus medius repair at Anne Arundel Medical Center in Annapolis, Maryland, from October 2015 through December 2025 were included.Patients were categorized into no-brace (n=86) and brace (n=76) groups based on whether a brace was ordered; compliance was not explicitly measured in this study. Demographics, comorbidities, and surgical details were retrospectively extracted from the electronic medical record (EMR). The primary outcome was differences in complications between groups, and all complications, both medical and surgical, were included. Additional outcomes included physical therapy utilization, assistive device requirements, emergency department returns and readmissions, and reoperations. Univariate analyses (two-sided independent samples t-tests and chi-squared or Fisher's exact tests) were performed to compare demographics, comorbidity burden (measured as Charlson Comorbidity Index (CCI) scores), and surgical details between groups. CCI scores were compared using a Mann-Whitney test due to the non-normal distribution. The CCI scores were interpreted as proposed by the original authors with 0 being healthy, 1-2 being mild comorbidity burden, 3-4 being moderate comorbidity burden, and 5 or greater being severe comorbidity burden [12]. Surgical details assessed included repair type (open vs. arthroscopic) and tear type (partial vs. complete). Outcomes at 30 days, six weeks, and six months postoperatively were then compared between groups using univariate analyses; risk ratios with 95% confidence intervals were included. Statistical significance was assessed at p<0.05. All statistical analyses were performed in RStudio (Posit PBC, Boston, Massachusetts, United States).

## Results

On average, patients were 56±13 years with a body mass index of 29.4±6.0 kg/m^2^ and a CCI score of 1.7±1.5, indicating a low comorbidity burden. One hundred and thirty-four patients (83%) were female, and 120 patients (74%) presented with partial tears. No significant differences in demographics or tear type were observed between groups. However, a greater percentage of patients in the no-brace group underwent open repairs (33.7% vs. 18.4%; p=0.028) (Table 1). 

**Table 1:** Demographics, comorbidities, and surgical details P-values <0.05 in bold; all data presented as n (%) or mean±standard deviation; t test statistic presented for continuous measures; χ^2^ test statistic for categorical measures. *Mann-Whitney U test (U test statistic) performed due to the non-normal distribution on Shapiro-Wilk testing; data presented as median (25th-75th percentile). BMI: body mass index; CCI: Charlson Comorbidity Index

Variable	No brace (n=86)	Brace (n=76)	Test statistic (t, U, or χ^2^)	P-value
Age (yrs)	56.1±13.0	55.9±12.0	0.09	0.926
BMI (kg/m^2^)	29.5±5.4	29.3±6.7	0.23	0.821
Female sex	74 (86)	60 (78.9)	1.42	0.233
CCI*	2.0 (1.0-2.0)	1.5 (1.0-2.0)	0.63	0.793
Open repair	29 (33.7)	14 (18.4)	4.84	0.028
Complete tear	26 (30.2)	16 (21.1)	1.77	0.183

Postoperatively, patients were followed for 6.0±2.8 months on average, with no significant difference in follow-up time between groups. One hundred and fifty-seven patients (97%) attended outpatient physical therapy, and 18 (11%) required an assistive device other than crutches (wheelchair, walker, or cane), again with no differences between groups. At 30 days postoperatively, both groups experienced similar rates of emergency department return (no brace: 2 (2.3%) vs. brace: 4 (5.3%); p=0.421) and readmission (no brace: 0% vs. brace: 2 (2.6%); p=0.219). At six weeks postoperatively, similar rates of falls (no brace: 2 (2.3%) vs. brace: 1 (1.3%); p=1.000) and other complications (no brace: 1 (1.2%) vs. brace: 2 (2.6%); p=0.601) were observed between groups. These trends remained consistent within six months postoperatively, as both groups experienced similar rates of falls (no brace: 4 (4.7%) vs. brace: 1 (1.3%); p=0.372) and other complications including deep vein thrombosis, re-tear, fibromyalgia flare, and fracture (no brace: 5 (5.8%) vs. brace: 5 (6.6%); p=0.840). Reoperation rates within six months were also similar between groups (no brace: 0% vs. brace: 2 (2.6%); p=0.219). The combined six-month complication and reoperation rate was also not significant between groups (10 (11.6%) vs. 9 (11.8%); p=0.966) (Table 2). 

**Table 2:** Clinical outcomes All data presented as n (%) or mean±standard deviation; t test statistic presented for continuous measures; χ^2^ test statistic for categorical measures. *Fisher's exact test. **Risk ratio not calculated due to the presence of a zero count in one group. PT: physical therapy; AD: assistive device; ED: emergency department; DVT/PE: deep vein thrombosis/pulmonary embolism

Outcome measure	No brace (n=86)	Brace (n=76)	No brace-to-brace risk ratio	Risk ratio with 95% confidence interval	Test statistic (t or χ^2^)	P-value
Attended outpatient PT post-op	82 (95.3)	75 (98.7)	0.97	0.92-1.02	1.50	0.221
Attended in-home PT post-op	2 (2.3)	0 (0.0)	N/A**	N/A**	N/A*	0.499*
Required AD > crutches	11 (12.8)	7 (9.2)	1.39	0.57-3.40	0.52	0.469
30-day ED return	2 (2.3)	4 (5.3)	0.44	0.08-2.35	N/A*	0.421*
30-day readmission	0 (0.0)	2 (2.6)	N/A**	N/A**	N/A*	0.219*
6-week fall	2 (2.3)	1 (1.3)	1.77	0.16-19.1	N/A*	1.000*
6-week other complication	1 (1.2)	2 (2.6)	0.44	0.04-4.78	N/A*	0.601*
6-month fall	4 (4.7)	1 (1.3)	3.53	0.40-30.9	N/A*	0.372*
6-month other complication	5 (5.8)	5 (6.6)	0.88	0.27-2.94	0.04	0.840
6-month other complication type						
DVT/PE	1 (1.2)	2 (2.6)	0.44	0.04-4.78	N/A*	0.601*
Re-tear	2 (2.3)	3 (4.0)	0.59	0.10-3.43	N/A*	0.666*
Other	2 (2.3)	0 (0.0)	N/A**	N/A**	N/A*	0.499*
6-month reoperation	0 (0.0)	2 (2.6)	N/A**	N/A**	N/A*	0.219*
Any 6-month complication or reoperation	10 (11.6)	9 (11.8)	0.98	0.42-2.29	0.00	0.966
Follow-up months	6.2±3.1	5.7±2.4	N/A	N/A	1.27	0.205

## Discussion

In this population, there were no significant differences in patient characteristics or outcomes between patients who used a hip abduction brace postoperatively and those who did not. Demographically, the groups were similar in age, body mass index, sex, CCI scores, and the number of patients with complete tears of the gluteus medius, but the group who did not wear a brace included more patients who underwent open repair. There were no significant differences in 30-day, six-week, and six-month complication rates between groups.

Gluteus medius tears can be repaired arthroscopically or using open surgical techniques. Historically, surgeons have opted for open repairs, but with increased training in hip arthroscopy and favorable outcomes, many gluteus medius tears are now repaired arthroscopically. Colvin et al. found that more fellowships are training surgeons to perform hip arthroscopy and advances in the technology allow the technique to be utilized for a wider range of pathologies [13]. Meghpara et al. reported that patient-reported outcome measures (PROMs) significantly improved from baseline two years after undergoing hip arthroscopy for gluteus medius tears [6]. Arthroscopy is beneficial because it is less invasive than traditional open surgery; however, the decision remains individualized based on surgeon experience. LaPorte et al. highlighted that arthroscopy minimizes damage to surrounding tissue but requires more training. Open surgery, on the other hand, allows for greater exposure of the tendon insertion on the greater trochanter and is less technically challenging but resulted in more postoperative complications [8]. In this study, more patients in the no-brace group underwent open surgery although similar complication rates were observed between groups. While not able to directly compare brace or no-brace protocols in open and arthroscopic surgeries separately, the similarity in complication rates between groups is encouraging from a safety standpoint. 

Postoperative recommendations regarding hip brace use after arthroscopy remain variable. Gupta et al. found that 59.2% of high-volume hip arthroscopy surgeons recommended postoperative brace use [11]. More recently, Bolia et al. surveyed expert surgeons (>500 cases) and non-expert surgeons, finding that 40% of the experts and 28% of non-experts prescribed postoperative brace use [14]. Although there is no literature on postoperative hip brace use after gluteus medius repairs, there are multiple studies on postoperative brace use for FAI. Johnson et al. found no difference in pain or complications between the brace and non-brace groups in a population of patients undergoing arthroscopic surgery for FAI [15]. Similarly, Wentzel et al. reported that hip brace use after arthroscopy did not improve PROMs or affect two-year reoperation rates [16]. In a randomized controlled trial, Hagen et al. found no difference in patient-reported outcomes, physical exam findings, physical function, and medication use in the brace vs. non-brace groups after surgery for FAI [17]. In alignment with FAI literature, this study saw no significant difference in complications without the use of a brace postoperatively. There is an opportunity for future research into the effects of postoperative bracing on PROMs and long-term functional outcomes following gluteus medius repair.

There may be some benefits from discontinuing the regular use of hip braces postoperatively. Braces on the market can cost up to $800, causing an economic burden on patients without a demonstrable reduction in complications with brace use. The anatomic location of the hip joint creates challenges for bracing, and patients have been shown to be dissatisfied with them due to discomfort, perceived ineffectiveness, and no improvement in pain or patient-reported outcomes [9,18]. With results showing no added benefit, the financial strain and dissatisfaction for patients can be avoided by discontinuing the routine prescription of a postoperative hip brace after gluteus medius repair.

There were several limitations to this study. First, this was a single-institution, single-surgeon observational retrospective study with a relatively small sample size, and there were very few complications observed in either group, highlighting the relatively low power in this study. Therefore, the negative findings of this study may be a result of a type 2 error and should be interpreted with caution. In addition, the use of historical controls due to the brace group being all prior to October 2020 and the no-brace group all after October 2020 may introduce some additional confounding factors, although the perioperative protocol remained the same throughout the study with the exception of the brace. There was a relatively short follow-up period of six months, although this time frame did include the period of time where patients would use a brace. Also, this study only reported clinical outcomes but did not measure functional or patient-reported outcomes, limiting the clinical relevance of our results. The significant difference in the rate of open versus arthroscopic procedures also may have affected the results, and future studies should examine these two groups independently. Finally, it is possible that unmeasured confounding factors influenced the results observed. Possible confounding variables not accounted for in the analysis include gluteus medius tear severity, concomitant procedures to surrounding structures, preoperative functional status, and brace compliance. Because the secondary outcomes analyzed were exploratory and several involved low event rates, p-values were not corrected for multiple comparisons; these results should therefore be interpreted as hypothesis-generating and warrant confirmation in larger cohorts. This study was not designed as a formal non-inferiority trial; treatment was not randomized, and no non-inferiority margin was pre-specified a priori. We therefore presented risk ratios with 95% confidence intervals rather than a non-inferiority analysis, allowing readers to evaluate both effect magnitude and precision directly. Non-significant comparisons should not be interpreted as evidence of equivalence, particularly given the low event counts and correspondingly wide confidence intervals for several outcomes. Future prospective studies with pre-specified non-inferiority margins and adequate power are needed to confirm these exploratory findings. 

## Conclusions

After the implementation of a no-bracing protocol, patients undergoing gluteus medius repair showed no significant difference in complications or reoperations at six months postoperatively and 30-day admissions or emergency department visits when compared to patients following a traditional bracing protocol. Discontinuing routine postoperative bracing following gluteus medius repair appears to be safe, with no increased complications in this group. Further study is required to validate these findings and determine the optimal postoperative protocol for this population.
